# Presence of *Babesia odocoilei* and *Borrelia burgdorferi* Sensu Stricto in a Tick and Dual Parasitism of *Amblyomma inornatum* and *Ixodes scapularis* on a Bird in Canada

**DOI:** 10.3390/healthcare7010046

**Published:** 2019-03-20

**Authors:** John D. Scott, Kerry L. Clark, Lance A. Durden

**Affiliations:** 1 International Lyme and Associated Diseases Society, 2 Wisconsin Circle, Suite 700, Chevy Chase, MD 20185-7007, USA; 2 Environmental Epidemiology Research Laboratory, Department of Public Health, University of North Florida, Jacksonville, FL 32224, USA; kclark@unf.edu; 3 Department of Biology, Georgia Southern University, Statesboro, GA 30458, USA; ldurden@georgiasouthern.edu

**Keywords:** *Borrelia burgdorferi* sensu stricto, Lyme disease, *Babesia*, babesiosis, 18S rRNA gene, ticks, birds, ectoparasite, tick-borne pathogens

## Abstract

Wild birds transport ticks into Canada that harbor a diversity of zoonotic pathogens. However, medical practitioners often question how these zoonotic pathogens are present in their locality. In this study, we provide the first report of an *Amblyomma inornatum* tick cofeeding with a blacklegged tick, *Ixodes scapularis*, which parasitized a Veery, *Catharus fuscescens*—a neotropical songbird. Using the flagellin (*flaB*) gene of the Lyme disease bacterium, *Borrelia burgdorferi* sensu lato, and the 18S rRNA gene of the *Babesia* piroplasm, a malaria-like microorganism, we detected *Borrelia burgdorferi* sensu stricto and *Babesia odocoilei*, respectively, in an *I. scapularis* nymph. After the molt, these ticks can bite humans. Furthermore, this is the first documentation of *B. odocoilei* in a tick parasitizing a bird. Our findings substantiate the fact that migratory songbirds transport neotropical ticks long distances, and import them into Canada during northward spring migration. Health care practitioners need to be aware that migratory songbirds transport pathogen-laden ticks into Canada annually, and pose an unforeseen health risk to Canadians.

## 1. Introduction

Ticks have medical and veterinary importance because they are vectors of multiple zoonotic microorganisms [1]. Hard-bodied ticks (Acari: Ixodidae) often harbor a wide array of bacterial, piroplasmic, and viral microorganisms, and transmit them to vertebrate hosts [1,2,3]. Wild birds provide long-distance dispersal of ixodid ticks, whereas terrestrial vertebrates facilitate short-distance movement. In the temperate zone of North America, Lyme disease and human babesiosis are the most frequent tick-borne diseases [4].

Lyme disease is a zoonotic disease caused by members of the *Borrelia burgdorferi* sensu lato (Bbsl) complex, and is generally transmitted to humans by blood-feeding ticks [5]. Worldwide, there are at least 23 genospecies in this complex, and *B. burgdorferi* sensu stricto is one of the genospecies that is pathogenic to humans. Not only do *Ixodes* species transmit Bbsl, certain *Amblyomma* and *Rhipicephalus* ticks have been reported to transmit Bbsl in east-central South America [6]. Morphologically, Bbsl is pleomorphic; it has diverse forms (i.e., spirochetes, blebs, round bodies, and granules) and, jointly, can form biofilms [7]. This spirochetal zoonosis has been associated with numerous dermatologic, neurologic, rheumatologic, cardiac, ophthalmological, otologic, gastrointestinal, endocrine, and psychiatric manifestations [8]. Only 14−41% of Lyme disease patients recall a tick bite [4,9], and 9−39% have an erythema migrans (EM) rash [4,10,11]; >50% have a homogeneous rash [10]. In nature, Bbsl is transmitted transstadially (larva to nymph to adult) in competent vector ticks, such as blacklegged ticks, *Ixodes scapularis* [1].

Human babesiosis is a zoonosis caused by members of the genus *Babesia*. These malaria-like microorganisms, which are piroplasms (Apicomplexa: Piroplasmida: Babesiidae), are typically transmitted by ixodid ticks to vertebrates, including humans. These apicomplexan parasites are highly adaptive in apposite ticks and suitable hosts. In *I. scapularis*, *Babesia* piroplasms are transmitted by transovarial transmission (female to eggs) [12] and by transstadial passage [13]. When a *Babesia*-infected tick starts to feed on a warm-blooded host, it transmits sporozoites from its salivary glands to the blood stream of its host, and sporozoites quickly invade erythrocytes. Patients may initially be asymptomatic, later have acute and fulminating symptoms that may culminate in death, especially if the patients are elderly (>55 years), splenectomized, immunocompromised, or coinfected with another tick-borne pathogen [13,14].

Songbirds (Passeriformes), in particular, can transport ticks hundreds of kilometers into Canada during northward spring migration [15,16,17,18,19,20,21,22]. Long-distance, passerine migrants can transport bird-feeding ticks from as far south as Brazil, and widely disperse them across Canada [20,21,22]. Whenever a songbird is parasitized by two or more tick species, which are infected with one or more pathogens, this avian host can become parasitemic and act as a reservoir of these tick-borne pathogens. 

In recent tick–host–pathogen studies, 15% to 59% of *I. scapularis* nymphs collected from songbirds in southern Canada were infected with Bbsl [17,20,21,22]. Additionally, immature stages of *Amblyomma* species, including *A. americanum*, *A. dissimile*, *A. humerale*, *A. imitator*, *A. inornatum*, *A. longirostre*, *A. maculatum*, *A. rotundatum*, and *A. sabanarae*, are imported into Canada by southern temperate and neotropical songbirds [23,24,25]. Passerine migrants have the potential to import a bevy of ticks and their associated tick-borne pathogens from the Neotropics. Zoogeographically, *A. inornatum* is primarily found in the southernmost Nearctic region, but also in the northern Neotropical region. The known natural range of *A. inornatum* extends from southern Texas to Panama [26,27]. This narrow landmass is a major flyway for migratory birds during bi-directional migration. In Canada, *A. inornatum* ticks were previously reported [17]; however, the background and descriptions for those ticks were incomplete. 

*Amblyomma inornatum* parasitizes a wide range of vertebrate hosts including Bovidae (e.g., sheep, goats, cattle), Cervidae (e.g., deer, elk), Dasypodidae (e.g., armadillo), Tayassuidae (e.g., peccary), Cricetidae (e.g., hispid cotton rats), Sciuridae (e.g., ground squirrels), Leporidae (e.g., rabbits, hares), Equidae (e.g., horses), Didelphidae (e.g., opossum), Felidae (e.g., cats), and Canidae (e.g., coyotes, dogs) [26,27,28,29,30]. This *Amblyomma* tick also parasitizes Galliformes (e.g., pheasants) and Passeriformes (perching birds, such as Veeries). From a medical standpoint, Medlin et al. state that *A. inornatum* bites humans [30]. 

The antiquity and origin of ticks and their associated pathogens is often questioned. Ancient ticks fossilized in amber have existed in the Western Hemisphere for more than 99 million years [31]. Some of these prehistoric ticks fed on feathered dinosaurs and certain ancient birds. Based on a fossilized tick from the Dominican Republic amber mines, *Babesia* piroplasms originated 30−45 Ma (million years ago) [32], while *Borrelia* bacteria date back 15−20 Ma [33]. Not only have the descendants of these ixodid ticks and associated microorganisms survived dramatic climate shifts, they have adapted to multiple arthropod–vertebrate interactions. 

Historically, on mainland Ontario, Banerjee et al. documented the first PCR-positive Bbsl isolate cultured from a blacklegged tick that was collected from an untraveled dog residing at Kenora [34]. Up to 56% of Lyme disease patients in the northeastern U.S.A. are concomitantly infected with *Babesia* piroplasms [35]. The intensity and profusion of symptoms in patients with simultaneous occurrence of Lyme disease and human babesiosis is normally greater than in patients with either disease alone [36]. The aim of this study was to determine whether passerines are continuing to transport extralimital ticks to Canada and to ascertain whether they are harboring any zoonotic pathogens that have not been identified previously. 

## 2. Materials and Methods

### 2.1. Tick Collection

Ticks were collected from a neotropical songbird by bird banders using fine-pointed, stainless steel forceps. Live ticks were put in a transparent, round-bottom, 8.5 mL polypropylene tube (15.7 mm × 75 mm, round based) (Sarstedt, Montréal, Québec, Canada). The mouth of the tube was covered with tulle netting (3-cm diameter) to allow ventilation for ticks. A polyethylene push cap with a 7-mm hole was placed into the mouth of the tube to secure the tulle netting, and prevent ticks escaping. Each tube, which contained the ticks from one host, was placed in a double-zipped plastic bag with a slightly moistened paper towel to maintain high humidity. All ticks were sent to the lab for identification (J.D.S.). The *Amblyomma* nymph was tentatively identified using a taxonomic key [27] and, following the nymph–adult molt, *Amblyomma* taxonomic keys for adults indigenous in the Western Hemisphere were used [28,37]. Similarly, for *Ixodes* nymphs, a nymphal taxonomic key was used [38]. *Ixodes* species were exposed to a long-day photoperiod of 16:8 h (Light:Dark), while *Amblyomma* ticks from the Neotropics were held at a photoperiod of 12:12 h (L:D). Complete records (i.e., geographic location, tick collection date, tick species, developmental life stage, and host species) were logged for each tick collection. To preserve ticks, they were stored in 2-mL microtubes containing 95% ethyl alcohol. The *Amblyomma* sp. female, 18-5A70B, was compared to specimens in the U.S. National Tick Collection.

### 2.2. Bacteria and Piroplasm Detection

Ticks were tested using a nested PCR that amplifies a portion of the flagellin (*flaB*) gene of Bbsl, with slight variations from a previously described protocol [39]. The primary PCR assay, which targets a 497-nt fragment of the *flaB* gene, used the following primers, 271F: 5′-AAG-GAA-TTG-GCA-GTT-CAA-TCA-GG-3′ and 767R: 5′-GCA-TTT-TCT-ATT-TTA-GCA-AGT-GAT-G-3′. The secondary (nested) PCR amplified a 437-nt internal fragment using primers, 301F: 5′-ACA-TAT-TCA-GAT-GCA-GAC-AGA-GG-3′ and 737R: 5′-GCA-TCA-ACT-GTA-GTT-GTA-ACA-TTA-ACA-GG-3′.

For *Babesia* testing and DNA sequencing of ticks, the same protocol was used as previously described by Casati et al. [40]. DNA sequencing of amplicons of the 18S ribosomal RNA (18S rRNA) of the genus *Babesia* was employed to delineate species. Of note, the 18S rRNA gene is a highly conserved gene.

### 2.3. DNA Sequence Analysis 

PCR products from the *Babesia* 18S rRNA and the *Borrelia* flaB positive samples were purified using the Wizard® SV Gel and PCR Clean-Up System (Promega, Madison, WI, USA). DNA templates were sequenced [41] using both the forward and reverse primers used in the nested PCRs. Investigator-derived sequences were compared with those obtained by searching the GenBank database (National Center for Biotechnology Information) using the Basic Local Alignment Search Tool (BLAST) [42], and aligned using Clustal X [43]. 

Nucleotide sequence accession numbers: The DNA nucleotide sequences for the *Babesia* 18S and Bbsl *flaB* gene fragments obtained from ticks in this study were deposited in the GenBank database with accession numbers MK628544 and MK620851, respectively.

### 2.4. Molecular Tick Identification 

The methodology used for the molecular identification of the *Amblyomma* sp. female, 18-5A70B, was carried out at the Centre of Biodiversity Genomics (CBG), University of Guelph. The algorithm was previously described [44].

## 3. Results

### 3.1. Identification of Amblyomma Tick

An *Amblomma* sp. nymph was collected from a Veery, *Catharus fuscescens*, on 16 May, 2018, at the Ruthven Park National Historic Site Banding Station, Haldimand Bird Observatory, Cayuga, Ontario: 42.97 N, 79.87 W. Using a photoperiod of 12:12 h (L:D), this fully engorged *Amblyomma* nymph molted to a female in 37 d. The newly formed female was allowed to become fully sclerotized before it was morphologically identified. The author (J.D.S.) provided preliminary identification, and the co-author (L.A.D.) provided verification of the identification. Taxonomic keys for adult *Amblyomma* species in the Neotropics were also employed to confirm the identification of this tick species [28,37]. This female specimen was also compared with confirmed *A. inornatum* females in the U.S. National Tick Collection, matched favorably with them, and the species was confirmed as *A. inornatum*. 

### 3.2. Identification of Tick-Borne Pathogens

Based on DNA sequence analysis, *B. burgdorferi* sensu stricto for Bbsl, and *B. odocoilei* were detected in the *I. scapularis* nymph.

### 3.3. Molecular Tick Assessment

Molecular analysis of the *Amblyomma* specimen (18-5A70B) was undertaken, but we were unable to amplify a valid barcode sequence for this tick specimen.

## 4. Discussion

We provide the first report of *A. inornatum* (nymph) and an *I. scapularis* (nymph) cofeeding on a bird in Canada. Even though these two tick species have individually been documented on passerines, this is the first-ever account of coinfestation on a bird anywhere. This discovery is congruent with previous bird-tick studies which show that migratory songbirds transport *Amblyomma* ticks from neotropical areas into Canada. Specifically, the *I. scapularis* nymph was coinfected with Bbsl and *B. odocoilei.* Ultimately, these tick-borne, zoonotic pathogens may be a serious health risk to humans.

### 4.1. Identification of Amblyomma inornatum

The coinfestation of *I. scapularis* and *A. inornatum* on a bird in Canada is a first. Even though Ogden et al. reported *A. inornatum* on wild birds in Canada, they did not provide details, such as background information, a morphological description, or confirmation of identification [17]. Since there are at least 57 *Amblyomma* spp. in the neotropical region, including the Caribbean sub-region, identifying larvae and nymphs morphologically makes for unreliable identifications [45]. Taxonomic keys are not available to delineate all of these immature species. When these larval and nymphal *Amblyomma* ticks are collected from spring migratory songbirds in Canada, there is a strong possibility for misidentification because they are outside their established geographical distribution. Moreover, the account of an *A. inornatum* male on a bird in the Ogden et al. study is questionable because adults are not known to parasite passerines. 

**Figure 1:**
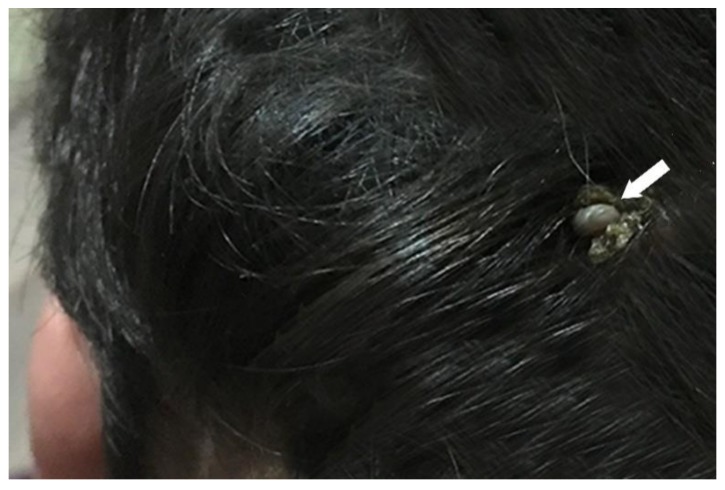
Blacklegged tick, *Ixodes scapularis*, partially engorged female parasitizing a 7-year-old boy. This female was attached on the nape of the neck where it had been feeding for 4 days. If this tick was infected with tick-borne pathogens, it could have easily transmitted tick-borne pathogens to the boy. Exudates formed around the tick when it was disturbed by hair brushing. If this female had become fully engorged, and was not found and removed, it could have caused tick paralysis (if it was genetically predisposed to synthesize paralysis biotoxins).

Within a tick-conducive microenvironment, replete nymphs (i.e., *I. scapularis*, *A. inornatum*) will molt to adults, and start questing for suitable vertebrate hosts, including humans. A photograph of an *I. scapularis* female parasitizing a human shows that ticks often take a blood meal in concealed locations on the body (Figure 1). 

In order to confirm the identification of *A. inornatum* in the present study, we allowed the fully engorged nymph to molt to an adult (female). After the nymph–adult molt, the *Amblyomma* female was keyed to *A. inornatum* using two *Amblyomma* keys for adults [28,37]. Morphologically, the distinguishing characteristics include: 1) Coxa I with an external spur that is much longer than the internal spur and 2) scutum inornate. The specimen matched favorably with confirmed *A. inornatum* females in the U.S. National Tick Collection. We provide confirmatory evidence of *A. inornatum* in Canada.

### 4.2. Flight Path of Veery 

Veeries start their northward migratory flight from their wintering grounds in central and southeastern South America. From there, they head northward to breeding grounds in north-central and northeastern U.S.A. These night-time fliers may use transoceanic pathways or employ overland routes through Central America [46]. Veeries can fly up to 285 km/night [47]. At this flight pace, a Veery could fly from the Mexico–U.S. border to Ruthven Park, Ontario, a distance of 2600 km, in 9 d. The Veery has a low percentage of body fat and, thus, must stop periodically along the flight path to replenish its energy reserves [47]. This timeline indicates that the *A. inornatum* nymph is a slow feeder during migratory flight. Moreover, a fully engorged tick normally drops from its host when the peripheral sensory organs deduce a suitable microhabitat [1]. Since *A. inornatum* is native to Central America, Mexico, and southern Texas, the Veery must have followed the Mississippi Flyway northward and proceeded through the Ohio River valley to Ruthven Park, Ontario.

In their indigenous distribution, *A. inornatum* nymphs have peak questing activity from February through May [30]. This host-seeking activity period corresponds with the northbound migration of Veeries en route through Central America. Likewise, the host-seeking activity period of *I. scapularis* nymphal ticks in the upper Midwest overlaps peak migration of Veeries [48]. Since the *I. scapularis* nymph was partially engorged, it most likely parasitized the Veery in the Ohio River valley. Both the fully engorged *A. inornatum* and the partially engorged *I. scapularis* were collected from the host bird at Ruthven Park in mid-May during peak spring migration, which coincides with the peak questing period of nymphs for both *A. inornatum* and *I. scapularis*. 

### 4.3. Sequence of Zoonotic Infection During Flight of Veery

The presence of Bbsl and *B. odocoilei* in the *I. scapularis* nymph, but not in the *A. inornatum* nymph, indicates that the Veery was likely not infected with these two pathogens. Based on the flight pace of the Veery and the extent of engorgement of the *I. scapularis* nymph, it is most likely that the host bird was parasitized in the Ohio valley two days before arrival and banding at Ruthven Park. The *I. scapularis* nymph possibly acquired Bbsl and *B. odocoilei* while taking a blood meal as a larva. More studies are needed to determine what vertebrate hosts simultaneously harbor both Bbsl and *B. odocoilei*. Even though *A. inornatum* is a non-indigenous tick in Canada, nymphs have ample time to molt during late spring and early summer to either males or females. Subsequently, both *A. inornatum* and *I. scapularis* females have adequate time to molt and transmit these tick-borne zoonotic microbes to humans.

In its native area, *A. inornatum* is known to harbor several tick-borne pathogens. For instance, Medlin et al. detected the following tick-borne pathogens in *A. inornatum* ticks collected in southern Texas: (1) endosymbiotic spotted fever group rickettsial species, *Rickettsia amblyommatis* (previously “*Candidatus Rickettsia amblyommii*”), (2) *Candidatus Borrelia lonestari* (a possible causative agent of southern tick rash-like illness), (3) *Ehrlichia chaffeensis* (causative agent of human monocytic ehrlichiosis), and (4) Bbsl [30]. 

When a pathogen-laden tick is cofeeding on a songbird with other ticks, it can transmit pathogens to these attached ticks. As soon as the host bird becomes parasitemic, it can systematically transmit pathogens to engorging ticks. For instance, *Babesia* sporozoites are stored in the tick salivary glands, and transmitted once the tick starts to take a blood meal from its host [12]. Endogenous transmission of sporozoites promptly occurs during the initial stage of engorgement. Not only can an infected bird be a reservoir, it can be an enzootic bridge for tick-borne pathogens. Whenever songbird-transported ticks are infected with one or more of these pathogens, they can subsequently induce acute illness in unsuspecting patients.

### 4.4. Tick–Host–Pathogen Dynamics

Cervids play an important role in the enzootic transmission cycle of *B. odocoilei*. The isolation of *B. odocoilei* from the blood of white-tailed deer, *Odocoileus virginianus*, was first achieved in Texas, and reveals that this cervid is a reservoir host [49,50]. Biogeographically, this piroplasm overlaps with the distribution of *I. scapularis* [50]. White-tailed deer are hosts of all three motile stages (larvae, nymphs, adults) of *I. scapularis*, and support the reproduction of this hematophagous ectoparasite. Ecologically, both white-tailed deer and *I. scapularis* perpetuate the enzootic transmission cycle of *B. odocoilei*. Transovarial and transstadial transmission of *B. odocoilei* occur in *I. scapularis* and, upon tick feeding, *Babesia* sporozoites are promptly transmitted, and entry ensues and multiplication takes place within the erythrocytes of cervid hosts [50]. Whenever larval or nymphal *I. scapularis* parasitize a white-tail deer, and become infected with *B. odocoilei*, they molt to the next life stage and can subsequently transmit sporozoites to vertebrate hosts, including humans. Paradoxically, Bbsl and *Babesia odocoilei* have different enzootic transmission pathways with cervid hosts. White-tailed deer are refractory to Bbsl, whereas cervids are reservoir-competent hosts of *B. odocoilei* [49,50]. 

With regard to avian hosts, Anderson and Magnarelli isolated Bbsl from the blood of passerine birds (i.e., Gray Catbird, Common Yellowthroat and American Robin) [51]. Pointedly, Anderson et al. isolated Bbsl from the liver of a Veery and, likewise, from *I. scapularis* larvae collected from Veeries [52]. In addition, McLean et al. cultured Bbsl from the blood of a Song Sparrow, *Melospiza melodia*, in the upper Midwest [53]. In the southeastern U.S.A., Durden et al. documented Bbsl in skin biopsies removed from passerine birds, and revealed that songbirds act as disseminators of Lyme disease spirochetes [54]. Richter et al. used spirochete-free xenodiagnostic *I. scapularis* larvae to show that the American Robin, *Turdus migratorius*, is a reservoir-competent host of Bbsl [55]. Since there is essentially no transovarial transmission of Bbsl in *I. scapularis* females [56], the *I. scapularis* nymph must have acquired Lyme disease spirochetes directly from the host Veery or indirectly when the nymph was feeding as a larva on a Bbsl-infected, reservoir-competent host. Pathologically, *I. scapularis* is known to carry and transmit at least 10 different tick-borne, zoonotic pathogens [1]. Upon repletion, some individual, fully engorged *I. scapularis* females can cause tick paralysis if they are not removed promptly.

In Europe, *Babesia microti* has been detected in *Ixodes ricinus* larvae feeding on European Robins, *Erithacus rubecula* [57]. In addition, *Babesia divergens* has been detected in bird-feeding *I. ricinus*, which is a competent vector of several tick-borne pathogens. In North America, Hersh et al. revealed that certain passerines (i.e., Wood Thrushes, Veeries, Gray Catbirds, American Robins) are reservoir competent hosts for *B. microti* [58]. They also documented a triple infection (i.e., *B. microti*, Bbsl, and *Anaplasma phagocytophilum*) in an *I. scapularis* nymph collected from a Veery. Based on our findings, and those of other researchers, Veeries play a significant role in the enzootic transmission dynamics of Bbsl and certain *Babesia* species.

Although there is uncertainty at this point in time, *B. odocoilei* may possibly be pathogenic to humans. This piroplasm is in the same sister group, namely the *Babesia* sensu stricto clade (i.e., EU1, a European genotype; *Babesia divergens; Babesia divergens*-like species) that is pathogenic to humans [59,60]. The EU1 strains are most closely related to *B. odocoilei*, but they differ significantly over the entire 18S rRNA gene from *B. odocoilei*. When *Babesia* serology is conducted, *B. odocoilei* may be cross-reacting with more familiar *Babesia* spp. (i.e., *Babesia microti*, *Babesia duncani*). More studies are needed to culture *B. odocoilei* from patients and to juxtapose them with *Babesia* serology.

### 4.5. Impact of Babesia and Borrelia burgdorferi Infections on Humans

Globally, there are over 100 recognized species of *Babesia* and, in North America, they infect a wide range of avian and mammalian hosts, including humans [61]. These apicomplexan piroplasms are pleomorphic, and have four diverse forms (i.e., gametes, sporozoites, merozoites, and trophozoites) [12]. When *Babesia*-infected ticks feed on vertebrates, sporozoites are transmitted from the tick’s salivary glands without delay, and invade the hosts’ red blood cells. Not only is this intraerythocytic parasite transmitted by ticks, it can be transmitted by blood transfusion [62] and transplacental transmission [63,64]. Common symptoms include fatigue, chills, sweats, headache, muscle aches, listlessness, dullness, nausea, and sleep disturbance. Cases of babesiosis can present with a high parasite burden, severe pathology, and fatal outcomes. Human babesiosis infections may recrudesce and persist and, ultimately, the resulting parasitemia can cause a fulminating, life-threatening zoonosis. Due to non-specific symptoms and possible coinfection with other tick-borne pathogens, human babesiosis is often misdiagnosed and underreported [65]. Although there are *Babesia* case reports in Canada, only one nationwide study of *B. duncani* has been published in the scientific literature [66].

The underdetection of Lyme disease and associated tick-borne diseases is a major pitfall across Canada [67]. The most frequently occurring clinical manifestations include neuro-associated symptoms (84%), fatigue (62%), and musculoskeletal-associated symptomology (57%) [4]. As a stealth pathogen, Bbsl slips by host defenses and sequesters in deep-seated tissue (i.e., ligaments, tendons, bone, eye, brain, muscle, glial and neuronal cells, synovium, and scar tissue) [68,69,70,71,72,73,74,75,76,77,78,79]. The persistence of Bbsl in chronic Lyme disease patients is well documented [4,80,81]. In the advanced stage, patients can have cognitive impairment that results in a gradually developing spectrum of neuropsychiatric symptoms [82,83]. When Lyme disease is not treated, or inadequately treated, chronic Lyme disease can result in fatal outcomes [72,84]. Transplacental transmission of Bbsl in humans has been documented with adverse fetal outcomes, including physiological dysfunction and musculoskeletal deformities [85,86,87,88,89]. 

Based on the MyLymeData, an online patient registry for chronic Lyme disease, 44% of the respondents had *Babesia* as the most common coinfection [4]. This study also revealed that psychiatric disorders (52%) were the most common misdiagnosis [4]. Concurrent Lyme disease and human babesiosis is typically more grave than either zoonosis by itself [35,90]. The co-occurrence of Lyme disease and human babesiosis typically makes clinical symptoms more severe, intense, and long-lasting [90,91]. Bbsl weakens the immune system, and makes these co-occurring infections more difficult to treat. 

## 5. Conclusions

We provide the first report of *B. odocoilei* in a tick parasitizing a bird. The presence of *B. odocoilei* and Bbsl in a bird-feeding *I. scapularis* nymph indicates that this vector tick may subsequently transmit a dual infection to a suitable host, including a human. The cofeeding of *A. inornatum* and *I. scapularis* ticks on a Veery is significant because it is the first-ever account of these two tick species simultaneously parasitizing a bird. This bird parasitism also reveals that neotropical songbirds can transport ticks long distances and introduce foreign pathogenic microbes from the Neotropics, Mexico, and the United States, and widely disperse them across Canada during northward spring migration. Since migratory songbirds transport multiple ticks and associated zoonotic pathogens from southern latitudes into Canada, health care practitioners must watch for, and fervently treat these tick-borne zoonoses with due diligence.

## References

[1] Nicholson W.A., Sonenshine D.E., Noden B.H., Mullen G.R., Durden L.A. (2019). Ticks (Ixodida). Medical and Veterinary Entomology.

[2] Reed K.D., Meece J.K., Henkel J.S., Shukla S.K. (2003). Birds, migration and emerging zoonoses: West Nile virus, Lyme disease, influenza A and enteropathogens. Clin. Med. Res..

[3] Goodman J.L., Dennis D.T., Sonenshine D.E. (2005). Tick–Borne Diseases of Humans.

[4] Johnson L., Shapiro M., Mankoff J. (2018). Removing the mask of average treatment effects in chronic Lyme disease research using Big Data and subgroup analysis. Healthcare.

[5] Burgdorfer W., Barbour A.G., Hayes S.F., Benach J.L., Grunwaldt E., Davis J.P. (1982). Lyme disease—A tick–borne spirochetosis?. Science.

[6] Miziara C.S.M.G., Serrano V.A.G., Yoshinari N. (2018). Passage of *Borrelia burgdorferi* through diverse ixodid hard ticks caused distinct diseases: Lyme borreliosis and Baggio–Yoshinari syndrome. Clinics.

[7] Meriläinen L., Herranen A., Schwarzbach A., Gilbert L. (2015). Morphological and biochemical features of *Borrelia burgdorferi* pleomorphic forms. Microbiology.

[8] Stricker R.B., Fesler M.C. (2018). Chronic Lyme disease: A working case definition. Am. J. Infect. Dis..

[9] Berger B.W. (1989). Dermatologic manifestation of Lyme disease. Rev. Infect. Dis..

[10] Stonehouse A., Studdiford J.S., Henry A. (2010). An update on the diagnosis and treatment of early Lyme disease: “focusing on the bull’s–eye, you may miss the mark”. J. Emerg. Med..

[11] Johnson L., Mankoff J., Stricker R.B. (2014). Severity of chronic Lyme disease compared to other chronic conditions: A quality of life survey. PeerJ.

[12] Mehlhorn H., Shein E. (1984). The piroplasms: Life cycle and sexual stages. Adv. Parasitol..

[13] Kjemtrup A.M., Conrad P.A. (2000). Human babesiosis: An emerging tick–borne disease. Austral. Soc. Parasitol..

[14] Homer M.J., Aguilar–Delfin I., Telford S.R., Krause P.J., Persing D.H. (2000). Babesiosis. Clin. Microbiol. Rev..

[15] Scott J.D., Fernando K., Banerjee S.N., Durden L.A., Byrne S.K., Banerjee M., Mann R.B., Morshed M.G. (2001). Birds disperse ixodid (Acari: Ixodidae) and *Borrelia burgdorferi*–infected ticks in Canada. J. Med. Entomol..

[16] Morshed M.G., Scott J.D., Fernando K., Beati L., Mazerolle D.F., Geddes G., Durden L.A. (2005). Migratory songbirds disperse ticks across Canada, and first isolation of the Lyme disease spirochete, *Borrelia burgdorferi*, from the avian tick, *Ixodes auritulus*. J. Parasitol..

[17] Ogden N.H., Lindsay L.R., Hanincová K., Barker I.K., Bigras–Poulin M., Charron D.F., Heagy A., Francis C.M., O’Callaghan C.J., Schwartz I. (2008). Role of migratory birds in introduction and range expansion of *I. scapularis* ticks and of *Borrelia burgdorferi* and *Anaplasma phagocytophilum* in Canada. Appl. Environ. Microbiol..

[18] Scott J.D., Lee M.-K., Fernando K., Durden L.A., Jorgensen D.R., Mak S., Morshed M.G. (2010). Detection of Lyme disease spirochete, *Borrelia burgdorferi* sensu lato, including three novel genotypes in ticks (Acari: Ixodidae) collected from songbirds (Passeriformes) across Canada. J. Vector Ecol..

[19] Scott J.D., Anderson J.F., Durden L.A. (2012). Widespread dispersal of *Borrelia burgdorferi*–infected ticks collected from songbirds across Canada. J. Parasitol..

[20] Scott J.D., Durden L.A. (2015). New records of the Lyme disease bacterium in ticks collected from songbirds in central and eastern Canada. Int. J. Acarol..

[21] Scott J.D., Clark K.L., Foley J.E., Bierman B.C., Durden L.A. (2018). Far–reaching dispersal of *Borrelia burgdorferi* sensu lato–infected blacklegged ticks by migratory songbirds in Canada. Healthcare.

[22] Scott J.D., Clark K.L., Foley J.E., Anderson J.F., Bierman B.C., Durden L.A. (2018). Extensive distribution of the Lyme disease bacterium, *Borrelia burgdorferi* sensu lato, in multiple tick species parasitizing avian and mammalian hosts across Canada. Healthcare.

[23] Scott J.D., Durden L.A. (2015). *Amblyomma dissimile* Koch (Acari: Ixodidae) parasitizes bird captured in Canada. Syst. Appl. Acarol..

[24] Scott J.D., Durden L.A. (2015). First record of *Amblyomma rotundatum* tick (Acari: Ixodidae) parasitizing a bird collected in Canada. Syst. Appl. Acarol..

[25] Scott J.D., Mahala G. (2015). Birds widely disperse pathogen–infected ticks. Seabirds and Songbirds: Habitat Preferences, Conservation, Migratory Behavior.

[26] Guglielmone A.A., Estrada–Peña A., Keirans J.E., Robbins R.G. (2003). Ticks (Acari: Ixodida) of the Neotropical Zoogeographic Region.

[27] Keirans J.E., Durden L.A. (1998). Illustrated key to nymphs of the tick genus *Amblyomma* (Acari: Ixodidae) found in the United States. J. Med. Entomol..

[28] Guzmán–Cornejo C., Robbins R.G., Guglielmone A.A., Montiel–Parra G., Pérez M. (2011). The *Amblyomma* (Acari: Ixodida: Ixodidae) of Mexico: Identification keys, distribution and hosts. Zootaxa.

[29] Guglielmone A.A., Robbins R.G., Apanaskevich D.A., Petnery T.N., Estrada–Pena A., Horak I.G. (2014). The Hard Ticks of the World (Acari: Ixodidae).

[30] Medlin J.S., Cohen J.I., Beck D.L. (2015). Vector potential and population dynamics for *Amblyomma inornatum*. Ticks Tick Borne Dis..

[31] Peñalver E., Arillo A., Delclòs X., Peris D., Grimaldi D.A., Anderson S.R., Nascimbene P.C., Pérez–de la Fuente R. (2018). Ticks parasitised feathered dinosaurs as revealed by Cretaceous ambler assemblages. Nature Commun..

[32] Poinar G. (2017). Fossilized mammalian erythrocytes associated with a tick reveal ancient piroplasms. J. Med. Entomol..

[33] Poinar G. (2015). Spirochete–like cells in a Dominican amber *Amblyomma* tick (Arachnida: Ixodidae). Hist. Biol..

[34] Banerjee S.N., Christensen C.I., Scott J.D. (1995). Isolation of *Borrelia burgdorferi* on mainland Ontario. Can. Com. Dis. Rep..

[35] Benach J.L., Coleman J.L., Habicht G.S., MacDonald A., Grunwaldt E., Giron J.A. (1985). Serological evidence for simultaneous occurrences of Lyme disease and babesiosis. J. Infect. Dis..

[36] Curcio S.R., Tria L.P., Gucwa A.L. (2016). Seroprevalence of *Babesia microti* in individuals with Lyme disease. Vector Borne Zoonotic Dis..

[37] Jones E.K., Clifford C.M., Kohls G.M. (1972). The ticks of Venezuela (Acarina: Ixodoidea) with a key to the species of Amblyomma in the Western Hemisphere.

[38] Durden L.A., Keirans J.E. (1996). Nymphs of the Genus Ixodes (Acari: Ixodidae) of the United States: Taxonomy, Identification Key, Distribution, Hosts, and Medical/Veterinary Importance. Monographs.

[39] Clark K., Hendricks A., Burge D. (2005). Molecular identification and analysis of *Borrelia burgdorferi* sensu lato in lizards in the southeastern United States. Appl. Environ. Microbiol..

[40] Casati S., Sager H., Gern L., Piffaretti J.-C. (2016). Presence of potentially pathogenic *Babesia* sp. for human in *Ixodes ricinus* in Switzerland. Ann. Agric. Environ. Med..

[41] McCombie W.R., Heiner C., Kelly J.M., Fitzgerald M.G., Gocayne J.D. (1992). Rapid and reliable fluorescent cycle sequencing of double stranded templates. DNA Seq..

[42] Altschul S.F., Gish W., Miller W., Myers E.W., Lipman D.J. (1990). Basic local alignment search tools. J. Mol. Biol..

[43] Thompson J.D., Gibson T.J., Plewniak F., Jeanmougin F., Higgins D.G. (1997). The ClustalX–Windows interface: Flexible strategies for multiple sequence alignment aided by quality analysis tools. Nucleic Acids Res..

[44] Scott J.D., Durden L.A. (2015). First report of a blacklegged tick, *Ixodes scapularis* Say (Acari: Ixodidae), parasitizing a raptor in Canada. Syst. Appl. Acarol..

[45] Guglielmone A.A., Robbins R.G. (2018). Hard Ticks (Acari: Ixodida: Ixodidae) Parasitizing Humans.

[46] eBird Primary Reference. https://help.ebird.org/customer/en/portal/articles/1006835–recommended–citation.

[47] The Virtual Nature Trail at Penn State New Kensington Species Pages. https://www.psu.edu/dept/nkbiology/naturetrail/speciespages/veery.html.

[48] Mannelli A., Kitron U., Jones C.J., Slajchert T.L. (1994). Influence of season and habitat on *Ixodes scapularis* infestation on white–footed mice in northwestern Illinois. J. Parasitol..

[49] Holman P.J., Waldrup K.A., Wagner G.G. (1988). *In vitro* cultivation of a *Babesia* isolated from a white–tailed deer (*Odocoileus virginianus*). J. Parasitol..

[50] Holman P.J., Madeley J., Craig T.M., Allsopp B.A., Allsopp M.T., Petrini K.R., Waghela S.D., Wagner G.G. (2000). Antigenic, phenotypic and molecular characterization confirms *Babesia odocoilei* isolated from three cervids. J. Wildl. Dis..

[51] Anderson J.F., Magnarelli L.A. (1984). Avian and mammalian hosts for spirochete–infected ticks and insects in a Lyme disease focus in Connecticut. Yale J. Biol. Med..

[52] Anderson J.F., Johnson R.C., Magnarelli L.A., Hyde F.W. (1986). Involvement of birds in the epidemiology of Lyme disease agent *Borrelia burgdorferi*. Infect. Immun..

[53] McLean R.G., Ubico S.R., Norton Hughes C.A., Engstrom S.M., Johnson R.C. (1993). Isolation and characterization of *Borrelia burdorferi* from blood of a bird captured in the Saint Croix River Valley. J. Clin. Microbiol..

[54] Durden L.A., Oliver J.H., Kinsey A.A. (2001). Ticks (Acari: Ixodidae) and spirochetes (Spirochaetaceae: Spirochaetales) recovered from birds on a Georgia barrier island. J. Med. Entomol..

[55] Richter D., Spielman A., Komar N., Matuschka F.-R. (2000). Competence of American Robins as reservoir hosts for Lyme disease spirochetes. Emerg. Infect. Dis..

[56] Rollend L., Fish D., Childs J.E. (2013). Transovarial transmission of *Borrelia spirochetes* by *Ixodes scapularis*: A summary of the literature and recent observations. Ticks Tick Borne Dis..

[57] Hildebrandt A., Franke J., Meier F., Sachse S., Dorn W., Straube E. (2010). The potential role of migratory birds in transmission cycles of *Babesia* spp., *Anaplasma phagocytophilum*, and *Rickettsia* spp.. Ticks Tick Borne Dis..

[58] Hersh M.H., Osfeld R.S., McHenry D.J., Tibbetts M., Brunner J.L., Killilea M.E., LoGiudice K., Schmidt K.A., Keesing F. (2014). Co–infestation of blacklegged ticks with *Babesia microti* and *Borrelia burgdorferi* is higher than expected and acquired from small mammal hosts. PLoS ONE.

[59] Herwaldt B.L., de Bruyn G., Pieniazek N.J., Homer M., Lofy K.H., Siiemenda S.B., Fritsche T.R., Persing D.H., Limaye A.P. (2004). *Babesia divergens*–like infection, Washington State. Emerg. Infect. Dis..

[60] Gorenflot A., Moubri K., Percigout E., Carey B., Schetters T.P. (1998). Human babesiois. Ann. Trop. Med. Parasitol..

[61] Shock B.C., Moncayo A., Cohen S., Mitchell E.A., Williamson P.C., Lopez G., Garrison L.E., Yabsley M.J. (2014). Diversity of piroplasms detected in blood–fed and questing ticks from several states in the United States. Ticks Tick Borne Dis..

[62] Villatoro T., Karp J.K. (2019). Transfusion–transmitted babesiosis. Arch. Pathol. Lab. Med..

[63] Cornett J.K., Malhotra A., Hart D. (2012). Vertical transmission of babesiosis from a pregnant, splenectomized mother to her neonate. Infect. Dis. Clin. Pract..

[64] Fox L.M., Winger S., Ahmed A., Arnold A., Chou J., Rhein L., Levy O. (2006). Neonatal babesiosis: Case report and review of the literature. Pediatr. Infect. Dis. J..

[65] Sherr V.T. (2005). Human babesiosis—An unrecorded reality: Absence of formal registry undermines it detection, diagnosis and treatment, suggesting need for immediate mandatory reporting. Med. Hypotheses.

[66] Scott J.D., Scott C.M. (2018). Human babesiosis caused by *Babesia duncani* has widespread distribution across Canada. Healthcare.

[67] Lloyd V.K., Hawkins R.G. (2018). Under–detection of Lyme disease in Canada. Healthcare.

[68] Häupl T., Hahn G., Rittig M., Krause A., Schoerner C., Schönherr U., Kalden J.R., Burmester G.R. (1993). Persistence of *Borrelia burgdorferi* in ligamentous tissue from a patient with chronic Lyme borreliosis. Arthritis Rheum..

[69] Müller M.E. (2012). Damage of collagen and elastic fibres by *Borrelia burgdorferi*—Known and new clinical histopathogical aspects. Open Neurol. J..

[70] Oksi J., Mertsola J., Reunanen M. (1994). Subacute multiple–site osteomyelitis cause by *Borrelia burgdorferi*. Clin. Infect. Dis..

[71] Preac–Mursic V., Pfister H.W., Spiegel H., Burk R., Wilske B., Reinhardt S. (1993). First isolation of *Borrelia burgdorferi* from an iris biopsy. J. Clin. Neuroophthalmol..

[72] Oksi J., Kalimo H., Marttila R.J., Marjamäki M., Sonninen P., Nikoskelainen J., Viljanen M.K. (1996). Inflammatory brain changes in Lyme borreliosis: A report on three patients and review of literature. Brain.

[73] MacDonald A.B. (2007). Alzheimer’s neuroborreliosis with *trans*–synaptic spread of infection and neurofibrillary tangles derived from intraneuronal spirochetes. Med. Hypotheses.

[74] Miklossy J. (2011). Alzheimer’s disease—A neurospirochetosis. Analysis of the evidence following Koch’s and Hill’s criteria. J. Neuroinflamm..

[75] Frey M., Jaulhac B., Piemont Y., Marcellin L., Boohs P.M., Vautravers P., Jesel M., Kuntz J.L., Monteil H., Sibilia J. (1998). Detection of *Borrelia burgdorferi* DNA in muscle of patients with chronic myalgia related to Lyme disease. Am. J. Med..

[76] Ramesh G., Borda J.T., Dufour J., Kaushal D., Ramamoorthy R., Lackner A.A., Philipp M.T. (2008). Interaction of the Lyme disease spirochete *Borrelia burgdorferi* with brain parenchyma elicits inflammatory mediators from glial cells as well as glial and neuronal apoptosis. Am. J. Pathol..

[77] Ramesh G., Santana–Gould L., Inglis F.M., England J.D., Philipp M.T. (2013). The Lyme disease spirochete *Borrelia burgdorferi* induces inflammation and apoptosis in cells from dorsal root ganglia. J. Neuroinflamm..

[78] Girschick H.J., Huppertz H.I., Rüssmann H., Krenn V., Karch H. (1996). Intracellular persistence of *Borrelia burgdorferi* in human synovial cells. Rheumatol. Int..

[79] Klempner M.S., Noring R., Rogers R.A. (1993). Invasion of human skin fibroblasts by the Lyme disease spirochete, *Borrelia burgdorferi*. J. Infect. Dis..

[80] Middelveen M.J., Burke J., Sapi E., Bandoski C., Filush K.R., Wang Y., Franco A., Timmaraju A., Schlinger H.A., Mayne P.J. (2015). Culture and identification of *Borrelia* spirochetes in human vaginal and seminal secretions. F1000Research.

[81] Embers M.E., Hasenkampf N.R., Jacobs M.B., Tardo A.C., Doyle–Meyers A., Philipp M.T., Hodzic E. (2017). Variable manifestations, diverse seroreactivity and post–treatment persistence in non–human primates exposed to *Borrelia burgdorferi* by tick feeding. PLoS ONE.

[82] Bransfield R.C. (2017). Suicide and Lyme and associated diseases. Neuropsychiatr. Dis. Treat..

[83] Bransfield R.C. (2018). Aggressiveness, violence, homocidality, homicide, and Lyme disease. Neuropsychiatr. Dis. Treat..

[84] Liegner K.B., Duray P., Agricola M., Rosenkilde C., Yannuzzi L.A., Ziska M., Tilton R.C., Hulinska D., Hubbard J., Fallon B.A. (1997). Lyme disease and the clinical spectrum of antibiotic responsive chronic meningoencephalomyelitides. J. Spir. Tick Borne Dis..

[85] Feder H., Wilson C.B., Nizet V., Maldonado Y., Remington J.S., Klein J.O. (2016). Borrelia infections: Lyme disease. Remington and Klein’s Infectious Diseases of the Fetus and Newborn Infant.

[86] Lavoie P.E., Lattner B.P., Duray P.H., Malawista A.G., Barbour R.C. Culture positive, seronegative, transplacental Lyme borreliosis infant mortality. Proceedings of the IV International Conference on Lyme Borreliosis.

[87] MacDonald A.B., No. 4, Johnson R.C. (1989). Gestational Lyme borreliosis: Implications for the fetus. Rheumatic Disease Clinics of North America.

[88] Horowitz R.I. (2003). Lyme disease and pregnancy: Implications of chronic infection, PCR testing, and prenatal treatment. Proceedings of the 16th International Scientific Conference on Lyme Disease & Other Tick–Borne Disorders.

[89] Trevian G., Stinco G., Cinco M. (1997). Neonatal skin lesions due to a spirochetal infection: A case of congenital Lyme borreliosis. Int. J. Dermatol..

[90] Grunwaldt E., Barbour A.G., Benach J.L. (1983). Simultaneous occurrence of babesiosis and Lyme disease. N. Engl. J. Med..

[91] Cameron D.J., Johnson L.B., Maloney E.L. (2014). Evidence assessments and guideline recommendations in Lyme disease: The clinical management of known tick bites, erythema migrans rashes and persistent disease. Expert Rev. Anti-Infect. Ther..

